# Probing phase transition in VO_2_ with the novel observation of low-frequency collective spin excitation

**DOI:** 10.1038/s41598-020-58813-x

**Published:** 2020-02-06

**Authors:** Raktima Basu, V. Srihari, Manas Sardar, Sachin Kumar Srivastava, Santanu Bera, Sandip Dhara

**Affiliations:** 1 Surface and Nanoscience Division, Indira Gandhi Centre for Atomic Research, Homi Bhabha National Institute, Kalpakkam, 603102 India; 2 High pressure and Synchrotron Radiation Physics Division, Bhabha Atomic Research Centre, Mumbai, India; 3 Materials Physics Division, Indira Gandhi Centre for Atomic Research, Kalpakkam, 603102 India; 4 Water and Steam Chemistry Division, Bhabha Atomic Research Centre Facilities, Homi Bhabha National Institute, Kalpakkam, 603102 India

**Keywords:** Phase transitions and critical phenomena, Raman spectroscopy

## Abstract

VO_2_ is well known for its first order, reversible, metal-to-insulator transition (MIT) along with a simultaneous structural phase transition (SPT) from a high-temperature metallic rutile tetragonal (R) to an insulating low-temperature monoclinic (M1) phase via two other insulating metastable phases of monoclinic M2 and triclinic T. At the same time, VO_2_ gains tremendous attention because of the half-a-century-old controversy over its origin, whether electron-electron correlation or electron-phonon coupling trigger the phase transition. In this regard, V_1-x_Mg_x_O_2_ samples were grown in stable phases of VO_2_ (M1, M2, and T) by controlled doping of Mg. We have observed a new collective mode in the low-frequency Raman spectra of all three insulating M1, M2 and T phases. We identify this mode with the breather (singlet spin excitation) mode about a spin-Pierls dimerized one dimensional spin ½ Heisenberg chain. The measured frequencies of these collective modes are phenomenologically consistent with the superexchange coupling strength between V spin ½ moments in all three phases. The significant deviation of Stokes to anti-Stokes intensity ratio of this low-frequency Raman mode from the usual thermal factor exp(*hʋ*/*K*_B_*T*) for phonons, and the orthogonal dependency of the phonon and spinon vibration in the polarized Raman study confirm its origin as spin excitations. The shift in the frequency of spin-wave and simultaneous increase in the transition temperature in the absence of any structural change confirms that SPT does not prompt MIT in VO_2_. On the other hand, the presence of spin-wave confirms the perturbation due to spin-Peierls dimerization leading to SPT. Thus, the observation of spin-excitations resulting from 1-D Heisenberg spin-½ chain can finally resolve the years-long debate in VO_2_ and can be extended to oxide-based multiferroics, which are useful for various potential device applications.

## Introduction

**Figure 1:**
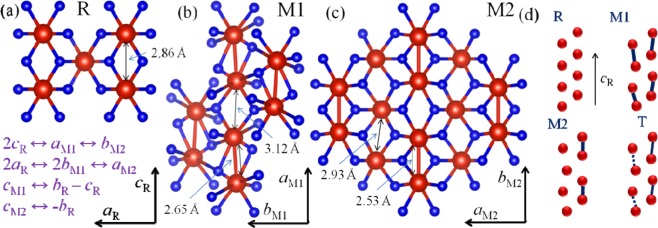
The schematic structures for (**a**) rutile (R), monoclinic (**b**) M1, and (**c**) M2 phases of VO_2_. Red and blue balls denote vanadium and oxygen atoms, respectively. (**d**) The arrangement of vanadium chains in the four phases without oxygen atoms.

The metal-to-insulator transition (MIT) in vanadium dioxide gains tremendous attention in scientific society because of its association with a structural phase transition (SPT). Close to room temperature (~ 340 K), VO_2_ undergoes a transition from low temperature insulating to a high-temperature metallic phase along with a structural transition from monoclinic, M1 (space group *P*2_1_/*c*) to rutile tetragonal R (space group *P*4_2_/*mnm*)^1,2^. In addition to M1 phase, another two insulating metastable phases of monoclinic, M2 (space group *C*2/*m*) and triclinic, T (space group $P\bar{1}$) are also accounted to evolve in the interim of the phase transition^3,4^. The phase stabilization of the metastable phases M2 and T are reported by introducing tensile strain^5,6^ along the rutile *c* axis (*c*_*R*_) by external mechanical means or by doping with metals of lower valency than V^4+^ ^7–9^. In both the insulating and metallic phases of VO_2_, there exist two parallel V-chains with each vanadium atom surrounded by six oxygen forming a distorted octahedron. The schematic structures of the various phases of VO_2_ are shown in Fig. 1.

The V chains are periodic and equally spaced with V-V separation of 2.86 Å in the high-temperature R phase (Fig. 1a)^10^. On the other hand, significant differences in the arrangement of V atoms along *c*_*R*_ axis are there in case of low-temperature monoclinic, M1, M2 and triclinic, T phases. The V atoms form pair (dimerized) alternately and tilt along the *c*_*R*_ axis in the M1 phase, which makes the unit cell volume double of that for the R phase with V-V separations of 2.65 (bonding) and 3.12 Å (anti-bonding) along *a*_M1_ ↔ 2*c*_R_ axis (Fig. 1b)^7,11^. In M2 phase, one set of V chains pair along the *c*_R_ axis without being twisted and the V-V separations being 2.53 Å (bonding) and 3.25 Å (anti-bonding) along *b*_M2_ ↔ 2*c*_R_ axis; while the V ions in the nearest neighbor V chains do not form pair but twist with respect to the *c*_R_ axis with V-V separation of 2.93 Å (Fig. 1c)^11^. The triclinic, T phase is reported as dimerized M2 phase Fig. 1d)^12^. Along with temperature, application of external electric field^13^, use of hydrostatic pressure^14^, radiance^15^, and applied deformation^16^, initiate phase transition in VO_2_. Furthermore, the transition temperature (*T*_c_) is varied by controlling the density of charge carrier^17^, invoking deformation^18^, or by doping^19^ leading to a considerable change in the optical, electrical, and thermal properties of VO_2_. These characteristics make VO_2_ an attractive material, namely, windows with heat control^20^, sensors for hazardous gas^21^, electrical switching device^22^, and cathode in Li-ion battery^23^. Doping of metal (M) in V_1-*x*_M_*x*_O_2_ reduces^24^ the *T*_c_ values for M = Ta^+5^, Nb^+5^, W^+6^, Mo^+6^, and increases^7–9^ the *T*_c_ values for M = Cr^+3^, Al^+3^, Ga^+3^.

As MIT is associated with SPT in this material, the debate remains, whether the electron-phonon coupling or strong electron-electron correlation trigger the phase transition^25,26^. In the present article, we study the cause of MIT and SPT and the correlation between them in details by our observation of collective spin excitation (spin-wave) for the first time in VO_2_. As spin-wave propagates independently from the charge-density waves (spin-charge separation according to Tomonaga-Luttinger liquid theory), the SPT and MIT are understood by separate phenomenological model. We claim that local strain due to Mg doping leads to the transformation of the M1 phase into M2 and T phases in the V_1-*x*_Mg_*x*_O_2_ system. Moreover, we claim that strong electron-electron correlation drives the MIT (Mott-Hubbard transition) and the V-chains in VO_2_ can be considered as one-dimensional (1-D) non-interacting Heisenberg spin ½ chains. The structural phase transition is understood by the observed low-frequency Raman modes originated due to the dimerized 1-D chains resulting from Mott transition at a lower temperature. The experimentally observed Raman modes at low-frequency are compared with the calculated frequency for the singlet breather excitations in spin-Pierls dimerized state. The orthogonal dependency of the phonon and spin excitation, in the polarized Raman study helps in finding the origin of the low-frequency Raman modes as spin singlet breather excitations. Moreover, it is found that the Stokes to anti-Stokes intensity ratio of the low-frequency Raman mode differs considerably from the Boltzmann’s distribution law for bosons confirming it to be originated due to collective spin excitation. The role of doping in shifting the frequency of spin-wave as well as in increasing the transition temperature while maintaining the same structural phase is discussed in details introducing finite-size 1-D Heisenberg spin ½ chain model resolving the years-long debate in the phase transition of VO_2_.

## Results and Discussions

**Figure 2:**
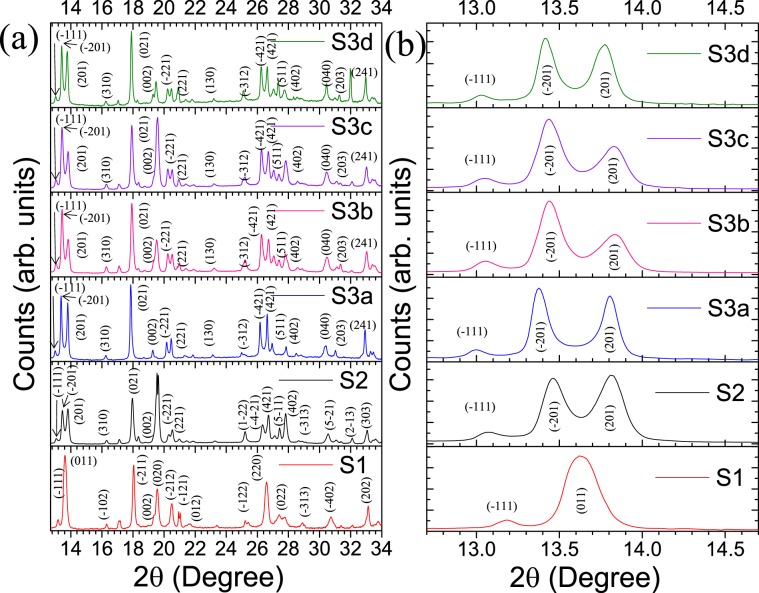
(**a**) The XRD pattern of the pristine samples S1, S2, and S3(a–d).The crystallographic (*hkl*) planes of the corresponding phases are indicated. (**b**) Zoomed image of the diffraction pattern for the lower angles.

The x-ray crystallographic studies of the ground free-standing samples with different Mg dopant are shown in Fig. 2a. In sample S1, the diffraction peaks confirm the presence of monoclinic M1 phase of VO_2_ (JCPDS # 04-007-1466)^27^. Whereas the diffraction peaks for sample S2 match with the T phase of VO_2_ (JCPDS # 01-071-0289)^28^. The samples S3 (a–d) are found out as the M2 phase of VO_2_ (JCPDS # 00-033-1441)^29^.

We focused on the diffraction peaks at lower 2θ values (Fig. 2b). For sample S1, the diffraction peak at 2θ = 13.67° represents the (011) plane corresponding to monoclinic M1 phase (equivalent to (110)_R_ plane) of VO_2_^27^. In sample S2, two diffraction peaks observed at 2θ = 13.47° and 13.81°may correspond to (−201) and (201) planes of T phase of VO_2_. Similarly, for sample S3(a–d) the diffraction peaks equivalent to (−201) and (201) planes of the M2 phase of VO_2_ are observed at ~ 2θ = 13.38° and 13.84°. The twin peaks in samples S2 and S3(a–d) are equivalent planes of (110)_R_ phase, which is responsible for twin crystalline formation. The T and M2 phases of VO_2_are reported as the strained (tensile strain along the rutile *c*_*R*_ axis) step for the M1 phase of VO_2_^6,30^. Since the samples were synthesized for different percentage of the Ar flow; there would be a different percentage of Mg dopants present in the samples. The dopant percentage can introduce strain in the sample and helps in stabilizing the T and M2 phases of VO_2_. All the samples in S3 series are found out to be stabilized in M2 phase of VO_2_, though the splitting of the twin peaks is observed to decrease from sample S3a (∆2θ = 0.42°) to S3d (∆2θ = 0.35°). A simultaneous decrease in the splitting of the twin peaks implies the increase in strain from sample S3a to S3d.

**Figure 3:**
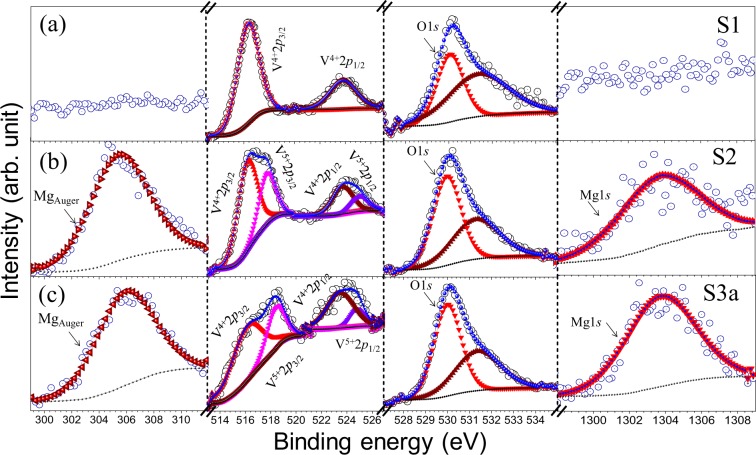
XPS spectra of the sample (**a**) S1, (**b**) S2, and (**c**) S3a denoted with the electronic transition of different elements. Open and solid symbols represent the data points and fitted curves, respectively.

In order to determine the dopant percentage in the samples, the x-ray photoelectron spectroscopy (XPS) studies were carried out for the samples S1, S2, and S3. The XPS spectra for different elements for sample S1 to S3 with the characteristic electronic transitions are shown in Fig. 3.

For sample S1, V 2*p* spin-orbit spectrum (Fig. 3a; 2^nd^ panel) can be fitted with two curves with binding energy (BE) values of 516.3 and 523.7 eV, which are assigned as the transition from 2*p*_3/2_ and 2*p*_1/2_ spin-orbits of V^4+^oxidization state, respectively^31,32^. No trace of Mg was observed in sample S1 (Fig. 3a; 1^st^ and 4^th^ panels). In case of sample S2, Mg 1s and Mg Auger peaks are observed at 1304 and 306.1 eV, respectively^33^, which are reported to be observed for the Mg ion bonded with O (Fig. 3b; 1^st^ and 4^th^ panels). The V 2*p* spin-orbit spectrum for sample S2 can be fitted by four peaks with BE values 516.2, 518.2, 523.6, and 524.8 eV (Fig. 3b; 2^nd^ panel). The V 2*p*_1/2_ and 2*p*_3/2_ peaks, observed at BE values of 516.2 and 523.6 eV, respectively for sample S2, can be assigned to V^4+^oxidization state. The peaks, observed at BE value of 518.2 and 524.8 eV, can be assigned to 2*p*_1/2_ and 2*p*_3/2_ transitions for V^5+^oxidization state^32^. In sample S3a, the peaks are identified at 516.3 and 523.7 eV for 2*p*_1/2_ and 2*p*_3/2_ transitions of V^4+^oxidization state, respectively. Similarly, for V^5+^ oxidization state, the peaks are identified at 518.2 eV and 524.8 eV as 2*p*_1/2_ and 2*p*_3/2_ transitions in sample S3 (Fig. 3c; 2^nd^ panel). The spin-orbit spectrum for Mg 1 *s* and Mg Auger peak, in the case of sample S3a, are observed at 1304 and 306 eV, respectively (Fig. 3b; 1^st^ and 4^th^ panels)^33^. On the other hand, for all the samples, O1*s* peak at low BE value (530 eV) is attributed to lattice O, and O1*s* peak with high BE value (531.5 eV) corresponds to absorbed O species (Fig. 3; 3^rd^ panel)^34,35^. The atomic percentage (at. %) of Mg, V, and O are calculated from the area under the curves considering appropriate sensitivity factors for each element and are tabulated (Table 1).

**Table 1:** The atomic percentage of the elements and V^5+^/V^4+^ratio calculated from XPS spectra.

Sample	V^4+^ (at. %)	V^5+^ (at. %)	O (at. %)	Mg (at. %)	V^5+^/V^4+^ ratio
S1	31.66	0	68.34	0	0
S2	21.42	8.19	69.57	0.81	0.38
S3a	19.77	8.50	69.75	1.97	0.43

For the samples S3(b–d), we observed nearly similar results as that of sample S3a in XPS analysis except for a small increase in at. % of Mg from sample S3a to S3d (not shown in the manuscript). We have performed Laser-induced breakdown spectroscopy (LIBS) to reconfirm the identification of the trace elements present in the samples (Supplementary Fig. S1). In the case of samples S3(a–d), the intensities of Mg lines are found to increase gradually (Fig. S1). The XPS and LIBS studies confirm the presence of Mg in samples S2 and S3(a–d), which may help in stabilizing the T and M2 phase of VO_2_, respectively, by introducing strain in the system (as discussed in crystallographic structural studies section). The replacement of V^4+^ (*d*^1^) by Mg ion is more likely to produce an adjacent V^+5^ (*d*^0^) sites in the neighboring chains. We have also carried out the x-ray absorption near edge structure (XANES) measurements to find out the oxidation state of the V in the samples (Supplementary Fig. S2). By comparing spectra, we confirm sample S1 is in +4 oxidation state, whereas samples S2 and S3 are in mixed +4 and +5 oxidation states.

**Figure 4:**
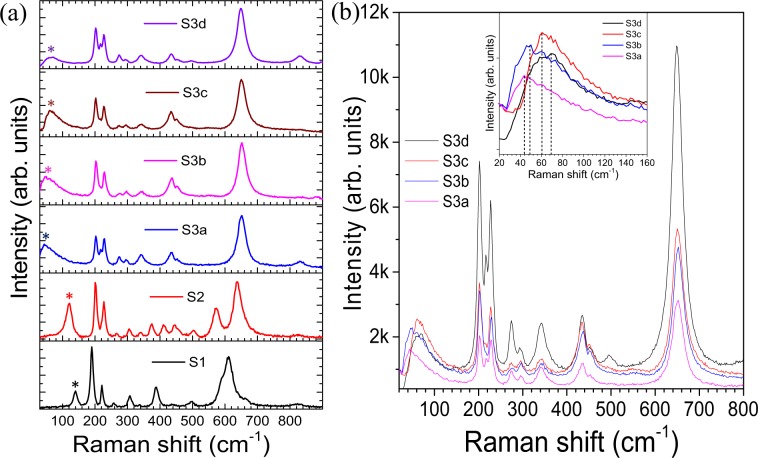
(**a**) Raman spectra of the samples S1 to S3 collected at 80 K and (**b**) Raman spectra for the samples S3(a–d). The inset shows a zoomed view of the Raman spectra for samples S3(a–d) at lower frequencies.

The Raman spectra of the samples were collected to obtain additional information about the structural phase of the as-grown samples. The Raman spectra for of S1, S2, and S3 samples collected at 80 K are depicted in Fig. 4a. Eighteen Raman-active phonon modes are predicted by Group theoretical analysis for all low-temperature phases of VO_2_ (for M1: 9*A*_g_ + 9*B*_g_, and for M2 and T: 10*A*_g_ + 8*B*_g_) at *Γ* point with different symmetries^14^. However, we observed twelve vibrational modes for sample S1 (Fig. 4a).

Observed Raman modes at 141, 190(*A*_g_), 225(*A*_g_), 258 (either *A*_g_ or *B*_g_,; *A*_g_/*B*_g_), 307(*A*_g_/*B*_g_), 335(*A*_g_), 390(*A*_g_/*B*_g_), 440(*A*_g_/*B*_g_), 497(*A*_g_/*B*_g_), 611(*A*_g_), 665(*B*_g_), 823(*B*_g_) cm^−1^ in sample S1(Fig. 4a) conforms with pure M1 phase of VO_2_^36,37^. In sample S2, thirteen mode frequencies are observed at 121, 201(*A*_g_), 228(*A*_g_), 267(*A*_g_/*B*_g_), 304(*A*_g_), 343(*A*_g_), 374(*A*_g_/*B*_g_), 413(*B*_g_), 445(*A*_g_/*B*_g_), 503(*A*_g_/*B*_g_), 572(*A*_g_), 636(*A*_g_), and 828(*B*_g_) cm^−1^ (Fig. 4a), which match with the reported data for T phase of VO_2_^4,17^. For samples S3(a–d) we observed eleven Raman modes at ~ 50, 203 (*A*_g_), 217(*A*_g_), 229(*A*_g_), 273(*A*_g_/*B*_g_), 297(*A*_g_), 341(*A*_g_), 432(*A*_g_/*B*_g_), 454(*A*_g_/*B*_g_), 651(*A*_g_), and 831(*B*_g_) cm^−1^ (Fig. 4a), which exactly resemble with the reported M2 phase of VO_2_^4,17^ Raman spectroscopic studies at 80 K confirm the presence of three different phases of VO_2_, i.e., M1, T, and M2 in samples S1, S2, and S3(a–d), respectively.

The Raman modes observed at 190 and 225 cm^−1^ for sample S1 are assigned to the vibration of V ions along and perpendicular to the *C*_R_ axis, respectively^4,12^. These modes are observed at 201 and 228 cm^−1^ for sample S2 and 203 and 229 cm^−1^ for sample S3. In the case of sample S3, one more Raman mode is also found to evolve at 217 cm^−1^. The shift in frequency for the Raman mode ~190 cm^−1^ for S1 → S2 → S3 transition is 13 cm^−1^, whereas the frequency shift for the mode ~225 cm^−1^ is only 4 cm^−1^. The above observations imply that doping of Mg introduces strain along *C*_R_ axis and thereby stabilizing the T and M2 phase of VO_2_ in samples S2 and S3(a–d), respectively. The Raman mode, observed at 611 cm^−1^ in sample S1, is due to V-O stretching vibration which shifts from 612 to 636 and 651 cm^−1^ in sample S2 and S3(a–d), respectively, indicating the length of V-O bond to be shorter with doping of Mg. In pure VO_2_, V^4+^ ion is positioned at the center of the octahedron constituting of oxygen atoms, and the principal axes of the octahedron are directed perpendicular to (110)_M1_ lattice plane^12^. Each V atom shared its four electrons with six neighbor O atoms, and each O atom attracted adjacent electrons supplying by three nearest V atoms. After Mg^2+^ ion occupies the native V^4+^ (+4) place, the adjacent V^+5^ (*d*^0^) sites replace V^4+^ (*d*^1^) sites in the neighboring chains, as observed from XPS (Fig. 3) and XANES (Fig. S2) studies. As the V^5+^ replaces V^4+^, two apical O^2−^ of the octahedron move closer to each other resulting reduction in the V-O bond length^38^. The 141 cm^−1^ band in sample S1 is reported as *A*_g_ mode in few of the earlier reports^37,39^. However, it is also reported as an external mode in one recent article^40^, which can be viewed as relative motions of structural units with respect to each other. The mode is observed at 121 cm^−1^ in sample S2 and ~50 cm^−1^ for samples S3(a–d) (denoted by “*” in Fig. 4a). The low-frequency Raman mode for samples S3(a–d) show a continuous blue-shift (inset of Fig. 4b) with an increase in doping while the other Raman peaks do not show any shift in frequency (Fig. 4b) with doping. VO_2_ is reported to consist of 1-D long Heisenberg chains of V ions in two adjacent sub-lattices^12,39^. As discussed before, in the M1 phase, the V atoms dimerize forming pairs, and the pairs tilt along the *c*_*R*_ axis in both the sub-lattices. Whereas, in case of the M2 phase, the V chains along the *c*_R_ axis pair without twisting in one sub-lattice, while in the other sub-lattice the V chains do not form pair. Instead, they twist with respect to the *c*_*R*_ axis. The dimerized V chains of M2 phase slightly twist in the intermediate (between the M1 and M2 phases) T phase. The electronic structures of VO_2_ were proposed by Goodenough in 1971^11^. In the electronic band structure of VO_2_, the O2*p* orbitals form the valence band with *π* and *σ* bonds and stay 2.5 eV below the Fermi level^41,42^. In VO_2_, there is single *d* electron per V atom and the *d* level splits into two states: the upper-lying doubly degenerate states ${e}_{g}^{\sigma }$states and lower-lying triply degenerate *t*_2*g*_ states. The *t*_2*g*_ states split again due to the tetragonal crystal field into an *a*_1*g*_ state (*d*_*xy*_) and an ${e}_{g}^{\pi }$ $({d}_{xz},\,{d}_{yz})$ doublet. In the low-temperature insulating phase of VO_2_, V atoms dimerize along the *c*_R_ direction which makes the *a*_1*g*_ bands to be split into lower (bonding, *a*_1*g*_) and upper (antibonding, *a*_1*g’*_) bands. Moreover, the twisting of the V-V pairs away from *c*_R_ axis enhances the V*d*-O*p* hybridization leading to a rise in the ${e}_{g}^{\pi }$ band above the Fermi level. As a result, a gap of ~0.7 eV opens up and makes the insulating phase stabilized^11,12^. However, in the high-temperature metallic phase, the Fermi level crosses partially filled *a*_1*g*_ and ${e}_{g}^{\pi }$ bands and the energy gap collapses. In this approach continuous changes in lattice parameters above transition temperature as well as sudden changes in lattice parameter due to V-V dimerization in the insulating phases is given as an input to calculate electronic structure, suggesting that it is the lattice that drives the MIT transition. In our previous report^39^, we argued a very different scenario for MIT. In the high temperature metallic phase three bands of different orbital characters overlap. The inter-orbital Coulomb repulsion pushes up the energy levels of the upper two bands, which leads to the transfer of electrons from the upper two bands to the lowest band. The electron transfer process leads to a further level of separation between bands. This process stops when two upper bands become empty, and the lowest band becomes half filled. The changes in lattice parameters above transition temperature are driven by the changes in relative band fillings with temperature. As soon as the lower band becomes half filled, the intra-orbital local Coulomb repulsion (Hubbard-type) takes over and drives a Mott-type MIT. The Mott insulating state is nothing but an assembly of parallel spin ½ antiferromagnetic Heisenberg chain. In 1-D, Heisenberg spin chain is driven to a spin-Pierls dimerized state.

In 1D spin ½ Heisenberg system, the Hamiltonian reads as,

1 $${H}_{0}=J{\sum }_{{\rm{j}}}{S}_{{\rm{j}}}.{S}_{{\rm{j}}+1}$$

where *J*(>0) is the exchange interaction between neighboring spins (*S*). The low-energy physics of this system is usually explained by a Tomonaga-Luttinger (TL) liquid with spinon excitation^43^. The Hamiltonian *H*_0_ and the corresponding Raman operator *R*_0_ commute with each other, and no Raman scattering is expected without additional perturbation. However, small perturbation ν_0_ exists due to spin-lattice coupling leading to the bond dimerization^44^. A perturbation due to static bond dimerizationis termed as,

2 $${\nu }_{0}={\sum }_{{\rm{j}}}J{(-1)}^{j}u{S}_{{\rm{j}}}.{S}_{{\rm{j}}+1}$$

where *u* is the distortion. Now the effective Hamiltonian becomes an exactly solvable Sine-Gordon (SG) model,

3 $$H^{\prime} ={H}_{0}+\int \frac{dx}{{a}_{0}}ud\,\sin (\sqrt{2\pi }\varphi )$$

where *x* = *ja*_0_; *a*_0_ is the lattice spacing, and *φ* is the canonical part of Bosonic fields.

There are three kinds of spin excitations; soliton (*S*; spinon), antisoliton (*S’*; anti-spinon), and some breathers (*B*_n_), which are the soliton-antisoliton (spinon-antispinon) bound states. The *n*th breather’s mass *E*_*n*_ is related to *E*_s_ (mass of soliton) via *E*_*n*_ = 2*E*_s_ sin [*nπ*/(8/*K*-2)]

With *n* = 1, …., [4/*K*-1]^45^. In SU(2)-symmetry, the soliton mass and second breather mass can be evaluated as

4 $${E}_{s} \sim 1.5{u}^{2/3}J,\,{\rm{and}}\,{E}_{2}=\sqrt{3}Es$$

Soliton, antisoliton and the first breather form a spin triplet with energy *E*_s_. The second breather is a singlet excitation. Raman scattering from 1-D spin ½ chain is theoretically explored by Sato *et al*.^44^. It is reported that in case of dimerized chain, Raman intensity due to the triplets gives rise to a continuum background at frequencies *ω* ≥ 2*E*_s_. Spin singlet breather *B*_2_ appears as a δ-functional peak at *ω* = *E*_2_ using form-factor approach (provided T < *J*)^46,47^. However, in a practical case, a broadening in δ-functional peak is expected as the experiment is carried out at non-zero temperature.

**Figure 5:**
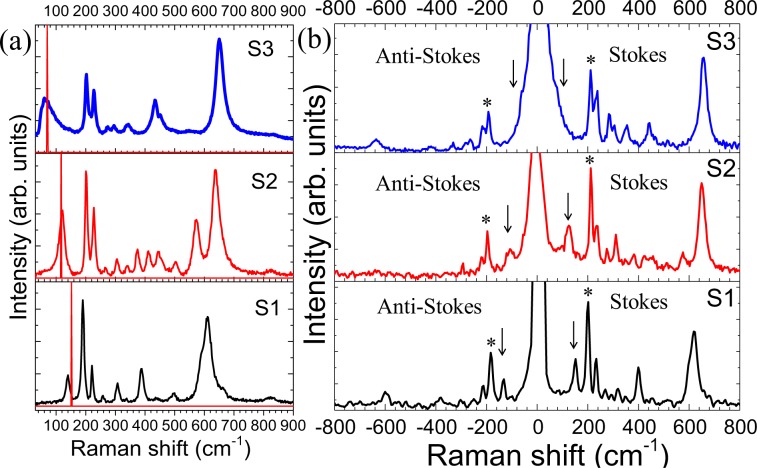
(**a**) Typical Raman spectra of all the samples S1 to S3a collected at 80 K and corresponding δ-functional peak at the calculated Raman mode frequency (**b**) Stokes and anti-Stokes spectra for the samples S1, S2, and S3. The spin-wave and the phonon mode used for calculation of (*I*_S_/*I*_AS_) ratio are denoted by arrow and star sign, respectively.

Pouget *et al*.^48^ reported the values of *J* for the different phases of VO_2_ by the nuclear magnetic resonance (NMR) and susceptibility studies. The value of *J* for the M1, T, and M2 phases of VO_2_ are found out as ~500 K, ~350 K, and ~320 K, respectively^48^. The distortion (*u*) dependence of Raman mode due to spin excitations is also reported for CuGeO_3_, TiOCr, and others^49–51^. We have calculated the distortion (*u* = ∆*l*/*l*, where *l* is the distance between two spins) for the three samples S1(M1), S2(T) and S3(M2) as *u*_1_ = 0.07, *u*_2_ = 0.08, and *u*_3_ = 0.12, respectively. The Raman mode frequencies for the samples in M1 (S1), T (S2), and M2 (S3) phases are calculated using Eq. (4) as *ω*_1_ = 152 cm^−1^, *ω*_2_ = 117 cm^−1^, and *ω*_3_ = 66 cm^−1^, respectively. The calculated and experimentally observed peaks are shown in Fig. 5a.

The little variation of experimental and calculated frequency is expected as the distortion *u* as well as exchange interaction *J* depends on doping concentration and temperature. Since the breather mode frequency is proportional to *u*^2/3^*J*, where *u* is the dimerization order parameter, its variation with temperature mimics temperature dependence of order parameter. A background luminescence is observed in the case of sample S3 (Fig. 5a), which may be due to the tilted non-dimerized and randomly dimerized V- chains as reported by Sato *et al*.^44^.

**Figure 6:**
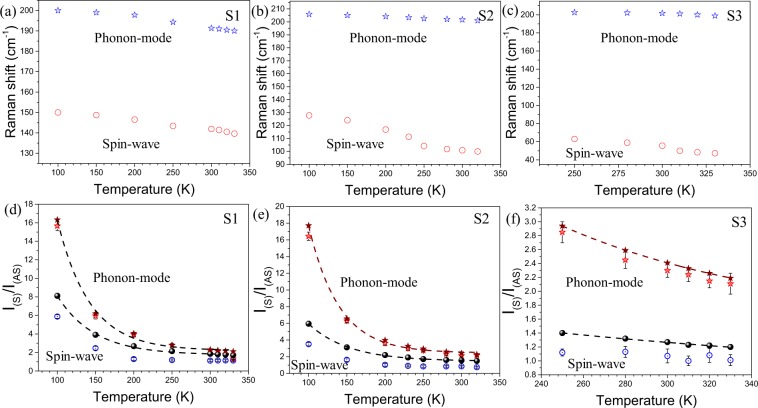
The frequency-shift with temperature for sample (**a**) S1, (**b**) S2, and (**c**) S3. The observed and calculated Stokes to anti-Stokes (*I*_S_/*I*_AS_) ratio with temperature for sample (**d**) S1, (**e**) S2, and (**f**) S3. The solid and empty symbols represent calculated and observed (*I*_S_/*I*_AS_) ratio, respectively, with corresponding error values.

To reconfirm the origin of low-frequency Raman modes, we have calculated the intensity ratio of the Stokes to anti-Stokes (*I*_S_/*I*_AS_) Raman spectra for both the spin-wave and phonon vibration as a function of temperature. The Stokes and anti-Stokes Raman spectra collected at room temperature are shown in Fig. 5b. The presence of spin-wave in both Stokes and anti-Stokes sides close to the Rayleigh line for all the three samples are denoted by an arrow mark (Fig. 5b). The frequency-shift as well as the integrated *I*_S_/*I*_AS_ ratio for the spinon mode at the lowest frequency and the closest phonon mode originated due to V-V vibration ~200 cm^−1^ (denoted by ‘*’ in Fig. 5b) are plotted as a function of temperature for all three samples (Fig. 6).

The *I*_S_/*I*_AS_ ratio is compared with the Boltzmann’s distribution law:

5 $$\frac{{I}_{S}}{{I}_{AS}}={(\frac{\upsilon -{\upsilon }_{m}}{\upsilon +{\upsilon }_{m}})}^{4}{e}^{\frac{h{\upsilon }_{m}}{{K}_{{\rm{B}}}T}}$$

where *ʋ* is the excitation frequency, *ʋ*_*m*_ is the phonon frequency, *h* is the Planck’s constant, *K*_B_ is the Boltzmann’s constant, and *T* is the temperature. The calculated values from Eq. (5) and experimentally observed values of *I*_S_/*I*_AS_ ratio for both the spin-wave and the closest phonon mode are tabulated (Table 2). The calculated *I*_S_/*I*_AS_ ratio are plotted in Fig. 6(d–f) as a function of temperature. It is clear from Fig. 6 that though there is a continuous red shift with an increase in temperature (Fig. 6(a–c)) for both the spin-wave and phonon mode; there is a significant mismatch between the observed *I*_S_/*I*_AS_ ratio and the calculated one for the spin-wave. However, *I*_S_/*I*_AS_ ratio for the closest phonon mode ~200 cm^−1^ follows the usual thermal factor of exp(*hʋ*/*K*_B_*T*) following Boltzmann’s distribution law.

**Table 2:** Comparison between calculated and experimentally observed *I*_S_/*I*_AS_ ratio for the three samples.

T(K)	Spin-wave			Phonon mode		
	ʋ (cm^−1^)	$\frac{{{\boldsymbol{I}}}_{{\boldsymbol{S}}}}{{{\boldsymbol{I}}}_{{\boldsymbol{AS}}}}$	${(\frac{{\boldsymbol{\upsilon }}-{{\boldsymbol{\upsilon }}}_{{\boldsymbol{m}}}}{{\boldsymbol{\upsilon }}+{{\boldsymbol{\upsilon }}}_{{\boldsymbol{m}}}})}^{{\bf{4}}}{{\boldsymbol{e}}}^{\frac{{\boldsymbol{h}}{{\boldsymbol{\upsilon }}}_{{\boldsymbol{m}}}}{{{\boldsymbol{K}}}_{{\boldsymbol{B}}}{\boldsymbol{T}}}}$	ʋ (cm^−1^)	$\frac{{{\boldsymbol{I}}}_{{\boldsymbol{S}}}}{{{\boldsymbol{I}}}_{{\boldsymbol{AS}}}}$	${(\frac{{\boldsymbol{\upsilon }}-{{\boldsymbol{\upsilon }}}_{{\boldsymbol{m}}}}{{\boldsymbol{\upsilon }}+{{\boldsymbol{\upsilon }}}_{{\boldsymbol{m}}}})}^{{\bf{4}}}{{\boldsymbol{e}}}^{\frac{{\boldsymbol{h}}{{\boldsymbol{\upsilon }}}_{{\boldsymbol{m}}}}{{{\boldsymbol{K}}}_{{\boldsymbol{B}}}{\boldsymbol{T}}}}$
***Sample S1***						
100	150 ± 0.1	5.88 ± 0.2	8.12	200 ± 0.1	15.66 ± 0.5	16.32
150	148.7 ± 0.1	5.47 ± 0.2	3.91	199 ± 0.1	5.97 ± 0.35	6.2
200	146.5 ± 0.1	1.79 ± 0.15	2.69	197.8 ± 0.1	3.94 ± 0.25	4.06
250	143.4 ± 0.1	2.19 ± 0.1	2.15	194.3 ± 0.1	2.78 ± 0.2	2.81
300	141.9 ± 0.1	1.62 ± 0.1	1.86	191.3 ± 0.1	2.20 ± 0.1	2.31
310	141.4 ± 0.1	3.44 ± 0.1	1.81	191 ± 0.1	2.13 ± 0.1	2.24
320	140.5 ± 0.1	2.95 ± 0.1	1.77	190.4 ± 0.1	1.81 ± 0.1	2.17
330	139.6 ± 0.1	1.74 ± 0.1	1.73	190 ± 0.1	1.31 ± 0.1	2.11
***Sample S2***						
100	127.8 ± 0.1	3.41 ± 0.2	5.95	205.9 ± 0.1	16.44 ± 0.5	17.72
150	124.1 ± 0.1	2.33 ± 0.2	3.12	205.1 ± 0.1	6.30 ± 0.25	6.55
200	116.9 ± 0.1	1.93 ± 0.15	2.20	204.2 ± 0.1	3.62 ± 0.25	3.98
230	111.3 ± 0.1	3.11 ± 0.1	1.91	203.4 ± 0.1	3.00 ± 0.2	3.27
250	102.2 ± 0.1	2.54 ± 0.15	1.72	202.6 ± 0.1	2.75 ± 0.1	2.94
280	101.8 ± 0.1	1.44 ± 0.1	1.61	202.1 ± 0.1	2.35 ± 0.15	2.59
300	100.9 ± 0.1	1.25 ± 0.1	1.55	201.7 ± 0.1	2.13 ± 0.1	2.41
320	100.0 ± 0.1	1.94 ± 0.1	1.50	201.1 ± 0.1	2.17 ± 0.1	2.27
***Sample S3***						
250	63.0 ± 0.1	1.02 ± 0.05	1.40	202.6 ± 0.1	2.85 ± 0.15	2.94
280	58.9 ± 0.1	1.03 ± 0.08	1.32	202.1 ± 0.1	2.45 ± 0.12	2.59
300	55.6 ± 0.1	1.07 ± 0.1	1.27	201.7 ± 0.1	2.30 ± 0.1	2.41
310	50.0 ± 0.1	1.00 ± 0.07	1.23	201.1 ± 0.1	2.24 ± 0.1	2.33
320	48.4 ± 0.1	1.18 ± 0.1	1.22	200.0 ± 0.1	2.15 ± 0.1	2.26
330	47.3 ± 0.1	1.11 ± 0.08	1.20	198.9 ± 0.1	2.11 ± 0.15	2.19

**Figure 7:**
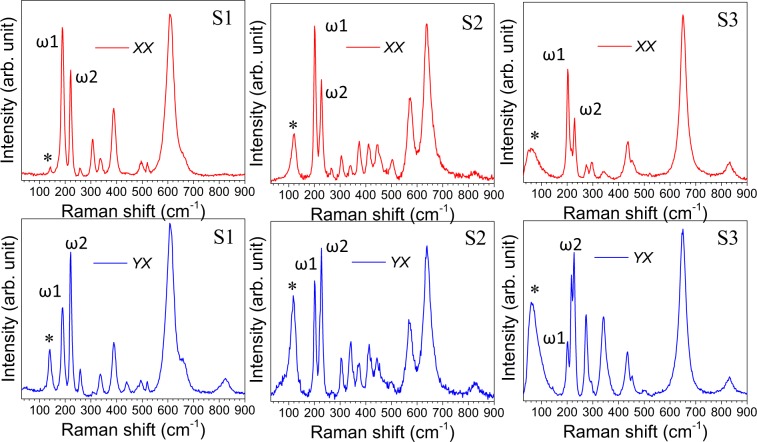
(**a**) Raman spectra at parallel (*XX*) and cross (*YX*) polarization condition for the three samples S1, S2, and S3.

Whereas, the lowest frequency mode for all the three samples shows a considerable deviation of *I*_S_/*I*_AS_ ratio from the usual thermal factor exp(*hʋ*/*K*_B_*T*) for phonons and thus confirming the mode to be originated from spin excitation^52,53^. We also performed the polarized Raman scattering on three single crystal microrods of the sample S1, S2, and S3. The FESEM image of the single microrod used for polarized Raman measurements along with randomly oriented microrods is shown in Supplementary Fig. S3a. The XRD data collected from a single microrod is also provided to confirm the orientation (Supplementary Fig. S3b). The incident excitation wave is considered along the *Z* direction, whereas, the growth direction of the microrods (*C*_R_ axis) is chosen along the *X*-axis. According to Porto notation, in the parallel $(Z(XX)\bar{Z})$ and perpendicular $(Z(YX)\bar{Z})$ polarization configurations, the first and fourth letters represent the direction of the propagation of the incident (***K***_i_) and scattered light (***K***_s_), respectively, whereas the second and third letters inside the parenthesis represents the direction of the electric field of the incident (***E***_i_) and the scattered light (***E***_s_), respectively. Figure 7 shows the Raman spectra with both parallel (*XX*) and cross (*YX*) polarization configurations for the three samples. In case of parallel (*XX*) polarization, the intensity of the Raman mode arising due to V-V vibration parallel to *C*_R_ axis (denoted as ω1 in Fig. 7) is more than that of the mode arising due to perpendicular to the *C*_R_ axis (denoted as ω2 in Fig. 7)^4,12,54^. Whereas, the intensity flips in case of cross (*YX*) polarization condition. The above observations imply the microrods are aligned along the X-axis, i.e., along the *C*_R_ direction^54^. However, the intensity of the low-frequency Raman-modes (denoted as ‘*’ in Fig. 7) for all the three samples is observed to be high in case of cross-polarization condition (⊥ to *C*_R_ axis), and falls rapidly for parallel polarization (|| to *C*_R_ axis). As the spin chains (V-ions) are aligned along the *C*_R_ direction, the spinon vibration is expected to be produced parallel to the *X-*axis.

In an electromagnetic (EM) wave, the electric field vector (***E***) and magnetic field vector (***B***) propagates orthogonally to each other. As the excitation EM wave was incident along *Z* direction; while the ***E*** field propagates along the *Y*-axis, the ***B*** field propagates along *X*-axis and vice-versa. The collective spins get excited by the magnetic part of the electro-magnetic wave; therefore the maximum intensity is expected for the cross-polarization (*YX*) condition where ***E*** and ***B*** field propagates along the *Y*-axis and *X*-axis, respectively. Thus the orthogonal dependency of the phonon and spin excitation in the polarized Raman study reconfirms the origin of the low-frequency Raman modes as spin excitation.

**Figure 8:**
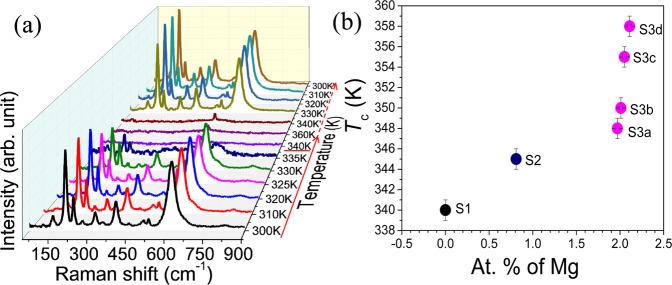
(**a**) Raman spectra of the sample S1 as a function of temperature. The transition temperature is underlined. Solid and dashed arrows denote the increase and decrease in temperature, respectively, and (**b**) Variation of transition temperature with at. % of Mg dopant.

We have carried out the temperature-dependent Raman spectroscopic analysis to study the phase transition of the V_1−x_Mg_x_O_2_ samples. The temperature dependent Raman data for all the three samples S1, S2 and S3b are shown in Supplementary Fig. S4. Above the transition temperature, VO_2_ became metallic in nature and we were unable to observe any Raman mode due to the screening effect. The temperature, at which sudden disappearance of all the Raman modes occurs, is considered as the transition temperature. We have also carried out temperature driven resistivity measurements (Supplementary Fig. S5) to reconfirm the transition temperatures. The resistance is observed to drop ~3 to 4 orders. Figure 8a shows typical temperature-dependent Raman spectra for sample S1. All the Raman modes disappear at 340 K for S1, 345 K for S2, 348 K for S3a, 350 K for S3b, 355 K for S3c, 358 K for S3d confirming the transition to metallic R phase^12,39^. The transition temperature is found to increase with an increase in Mg dopant (Fig. 8b).

Doping of Mg leads to sites with V^5+^ or *d*^0^ states (holes in the electron chains). The *d*^0^ sites effectively cut down the length of the infinitely long 1-D spin chains into smaller fragments^39^. The average size (*L*) of the spin-chains decreases with increase in *d*^0^ states, i.e., with an increase in Mg dopant. The temperature of Spin-Peierls transition in the infinite-sized system is lesser than that of in the finite-sized system. Takayoshi *et al*.^45^ reported for 1D spin ½ Heisenberg chains that the finite energy gap increases linearly with 1/*L* (finite chain length ~ *L*). As the doping concentration of Mg increases from samples S3a to S3d, the average length of finite-sized spin ½ Heisenberg chains decreases, which in turn increases the factor 1/*L*. The increase in 1/*L* results in an increase in *E*_s_ in SU(2) systems and thereby the Raman mode frequency *ω* increases as observed in Fig. 4b (*ω* = *E*_2_ and *E*_2_ = $\sqrt{3}$*E*_s_). The blue shift (inset of Fig. 4b) with an increase in doping is observed only for the low-frequency spinon mode, while the other vibrational modes remain unchanged (Fig. 4b) with doping. The variation in *T*_c_ with hole doping while maintaining the same monoclinic, M2 symmetry (Sample S3(a–d)) confirms that SPT does not have any role in MIT. Spin-Pierls dimerized state comes from, spin-phonon coupling in Heisenberg spin ½ antiferromagnetic chain. Heisenberg spin chain presupposes a Mott transition in a half filled band. It is to be emphasized that this spin-mode is also observed in M2 phase (insulating) where only half the V-V chains are dimerized, and the other chains are not dimerized. The fact that the un-dimerized chains are not electrically conducting suggests the metal-insulator transition is a correlation driven Mott-transition which prompts a simultaneous SPT with reduction of temperature.

## Conclusions

V_1-x_Mg_x_O_2_ samples were grown in stable phases of VO_2_ (M1, M2, and T) by controlled doping of Mg. Random local strain due to Mg doping is found out as the main cause for the evolution of the M1 phase into T and M2 phases. The unrelaxed local strain in the metastable phases, however, does not bring metallicity in the system. It is argued that strong electronic correlation drives the MIT and in turn, the Mott type MIT prompts a structural transition of spin-Peierls originating at a lower temperature. The insulating phases of VO_2_ can be considered as infinitely long 1-D dimerized Heisenberg spin ½ chains. The newly observed collective modes in the low-frequency Raman spectra of all three insulating M1, M2 and T phases are explained by the breather (singlet spin excitation) mode about a spin-Pierls dimerized 1-D spin ½ Heisenberg chain. The orthogonal dependency of the phonon and the singlet breathers in the polarized Raman study help in finding the origin of the low-frequency Raman modes as spin excitations. Moreover, it is found that the Stokes to anti-Stokes intensity ratio of the low-frequency Raman mode differs considerably from the Boltzmann’s distribution law confirming its origin from spin excitations. The fact that the M2 phase is insulating even though half the V-V chains are not dimerized conclusively proves that MIT is a Mott-Hubbard transition. The V vacancy, invoked by Mg doping, creates *d*^0^ sites (V^5+^) at the nearest neighbors and introduces finite-size scaling effect by reducing the effective length of the Heisenberg spin ½ chains. Thus, the role of doping in increasing the transition temperature is understood by introducing finite-size 1-D Heisenberg spin ½ chain model. As spin-wave propagates independently from the charge-density waves, the shift in the frequency of spin-wave with doping in the absence of any structural phase transition confirms that the SPT does not prompt the MIT and resolves the years-long debate in the phase transition of VO_2_.

## Methods

V_1−*x*_Mg_*x*_O_2_ microrods were grown by vapor transport process on high pure (99.99%) alumina boat using mixed VO_2_ powder and Mg powder (Sigma-Aldrich, 99%) as the source and Ar (99.9%) as the carrier gas. The synthesis was carried out at 1100 K for 3 h. The concentration of the Mg dopant was controlled by changing the flow rate of the carrier gas. The synthesis was carried with the optimized flow of Ar (99.9%), e.g., 20, 40, 60, 80, and 100 sccm (samples, S2, S3a, S3b, S3c, and S3d, respectively) in the presence of Mg powder. Sample S1 was prepared with the flow of 20 sccm Ar, keeping other growth conditions same except the Mg powder. The XPS (VG ESCALAB MK200X) analysis was performed for the elemental analysis of the VO_2_ samples synthesized at different growth conditions using an x-ray source of Al-Kα (1486.6 eV) with beam diameter around 3 mm. The C 1 *s* reference peak was used for calculating the BE values. A mixture of Gaussian–Lorentzian line shapes was used for fitting the spectra after applying Shirley type background correction. The structural properties of the as-grown samples were studied using synchrotron x-ray diffraction with a wavelength of 0.76089 Å using Si(111) channel cut monochromator. A MAR345 image plate area detector was used to collect the diffraction data. The vibrational modes of the synthesized samples were studied using a micro-Raman spectrometer (inVia, Renishaw, UK) in the backscattering configuration. An Ar^+^ Laser (514.5 nm) was used as the excitation source along with a thermoelectrically cooled CCD camera as the detector. To carry out the polarized Raman studies, polarizer and half-wave plates were placed in the incident ray path to attain the desired configurations. The low-frequency Raman scattering measurements for the Stokes and anti-Stokes spectra were carried out using a micro-Raman spectrometer (Witec Alpha 300RA) equipped with the Bragg grating (Rayshield™). Nd-YAG laser source (532 nm) was used to excite the samples. The temperature-dependent Raman spectra were collected using a temperature-controlled stage (Linkam; THMS600) under long working distance 50X objective with a numerical aperture (N.A.) of 0.45.

## Supplementary information

Supplementary information.
