# Supplementary material for: Probing phase transition in VO2 with the novel observation of low-frequency collective spin excitation
Source: Sci Rep. 2020 Feb 6;10:1977. doi: 10.1038/s41598-020-58813-x (PMC7005027; doi:10.1038/s41598-020-58813-x)
Supplement: Supplementary file 1 — Supplementary information. [file 41598_2020_58813_MOESM1_ESM.pdf]

Supplementary information

Probing phase transition in VO_2 with the novel observation of low-frequency collective spin excitation

Raktima Basu,[1,*] V. Srihari,[2] Manas Sardar,[3] Sachin Kumar Srivastava,[3] Santanu Bera,[4] and Sandip Dhara[1,*]

[1]Surface and Nanoscience Division, Indira Gandhi Centre for Atomic Research, Homi Bhabha National Institute, Kalpakkam-603102, India

[2]High pressure and Synchrotron Radiation Physics Division, Bhabha Atomic Research Centre, Mumbai, India.

[3]Materials Physics Division, Indira Gandhi Centre for Atomic Research, Kalpakkam-603102, India

[4]Water and Steam Chemistry Division, Bhabha Atomic Research Centre Facilities, Homi Bhabha National Institute, Kalpakkam-603102, India

[*] *Email: raktimabasu14@gmail.com; dhara@igcar.gov.in*

Laser induced breakdown spectroscopy (LIBS) is used for the identification and quantification of the elements present in the samples. The plasma emission was produced in an air atmosphere using a Q-switched Nd:YAG laser (Quantel, Brilliant) operated at 532 nm, with a pulse duration of 8–10 ns and repetition rate of 10 Hz. The laser beam was focused at the target surface by a plano-convex quartz lens of focal length 15 cm; producing a spot size of about 400 μm in diameter. Plasma emission was collected at an angle by an optical fibre of 600 μm aperture (LTB, Germany), coupled to a fused silica collimator with a focal point of 8.7 mm and imaged onto the Aryelle 200 spectrometer (LTB, Germany). The dispersed light was detected by a CCD detector

coupled to the spectrometer. We have performed LIBS to reconfirm the identification of the trace elements present in the samples. Figure S1 shows the LIBS spectra for the samples S1 to S3 (a-d). In sample S1 there is no trace for Mg, whereas, in sample S2, the emission lines of Mg (II) at 279.55 and 280.27 nm proves the presence of Mg in the sample (1). In the case of samples S3a to S3d, the intensity of Mg lines increases simultaneously (Figure S1). The V lines were identified between 437 to 441 nm for all the samples (2). The LIBS study confirms the presence of Mg in samples S2 and S3 (a-d), which may help in stabilizing the T and M2 phase of VO_2. As Mg (+2) has valency less than V (+4), the replacement of V^{4+} (d^1) by Mg ion is more likely to produce an adjacent V^{+5} (d^0) sites in the neighboring chains.

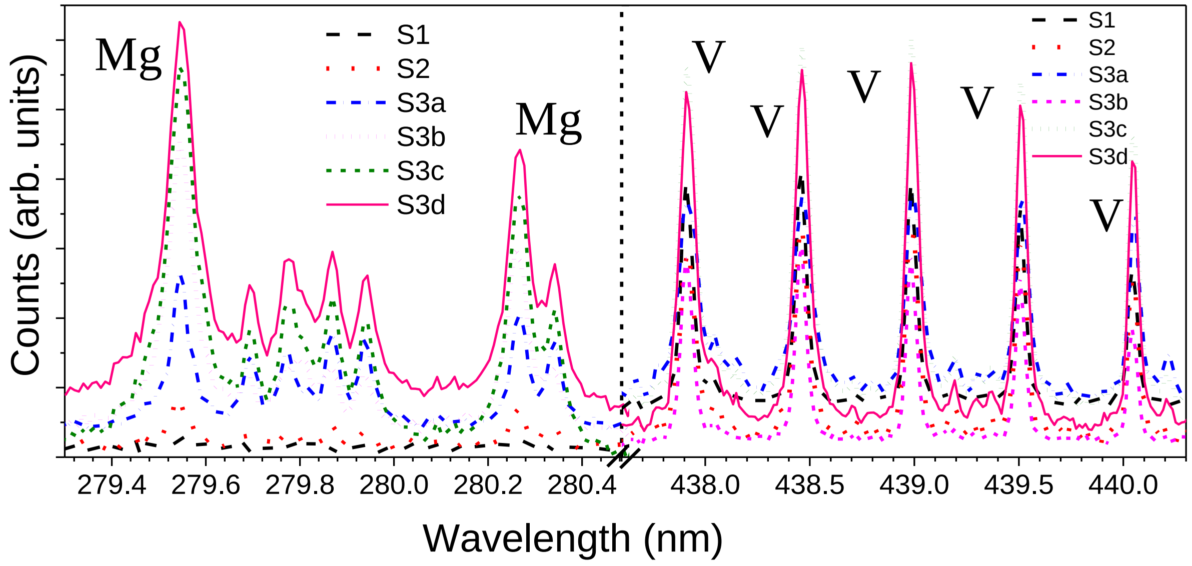

Fig. S1 LIBS spectra for the samples S1 to S3 (a-d) showing peaks for V and Mg in VO_2 matrix.

The x-ray absorption near edge structure (XANES) measurement was performed at scanning EXAFS beamline (BL-9) of Indus-2 synchrotron source at Vanadium K-edges in a transmission mode by putting the powder sample sandwiched between the scotch tapes. The background

removal and data normalization were done using ATHENA software. The background removed EXAFS data were fitted using ARTEMIS software.

We have carried out the XANES measurements to find out the oxidation state of the V in the samples (Figure S2). By comparing spectra, we can see sample S1 is in +4 oxidation state as the spectrum matches with that of standard VO_2 foil. Whereas samples S2 and S3 are in mixed +4 and +5 oxidation states, as the spectra go with the standard V_2O_5 foil also.

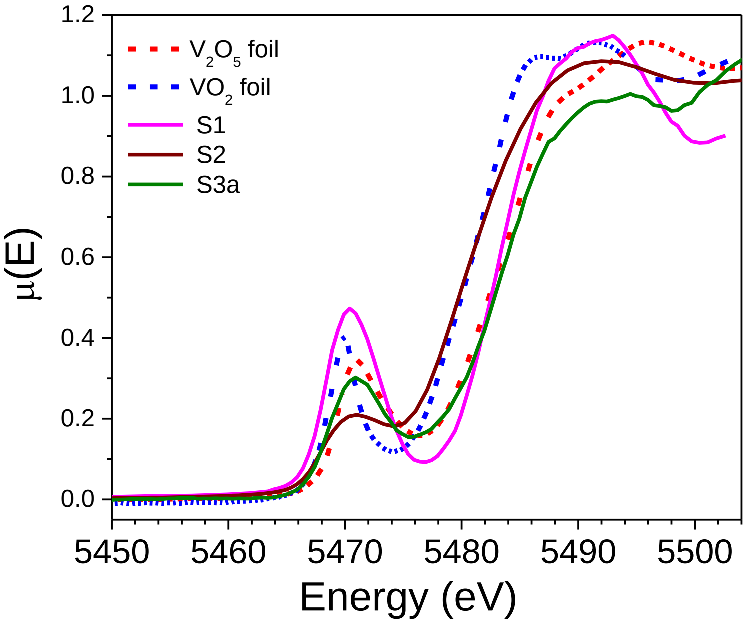

Fig. S2 Normalized XANES spectra at V K-edge for the samples S1 to S3 (solid lines). The XANES spectra for slandered VO_2 and V_2O_5 foils are also shown for comparison (dotted lines).

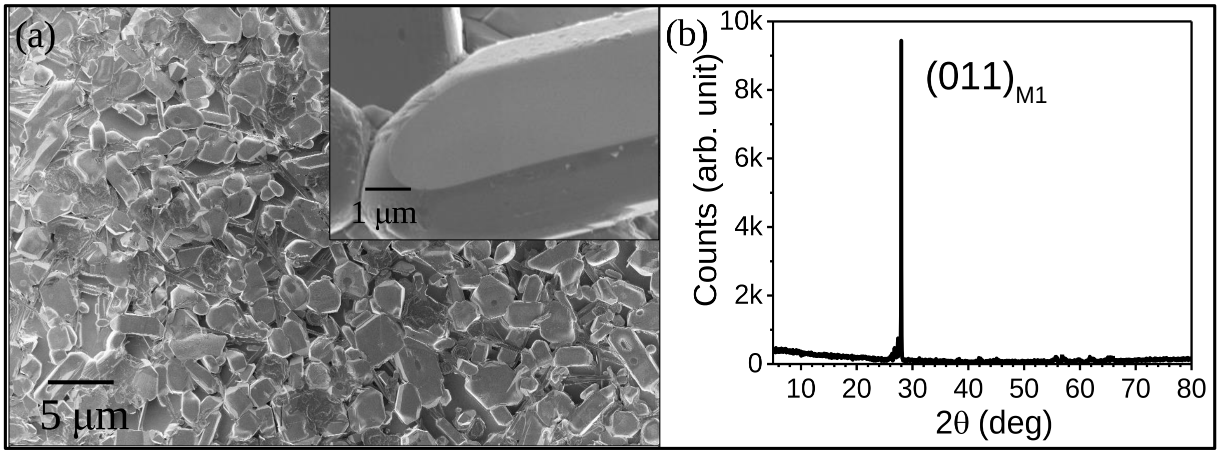

Fig. S3 (a) FESEM images of as-grown micro rods. Insets show magnified images of a single micro rods of size ~2 µm. (b) The GIXRD pattern collected from a single microrod using Cu Kα radiation source of wavelength, λ=1.5406 Å.

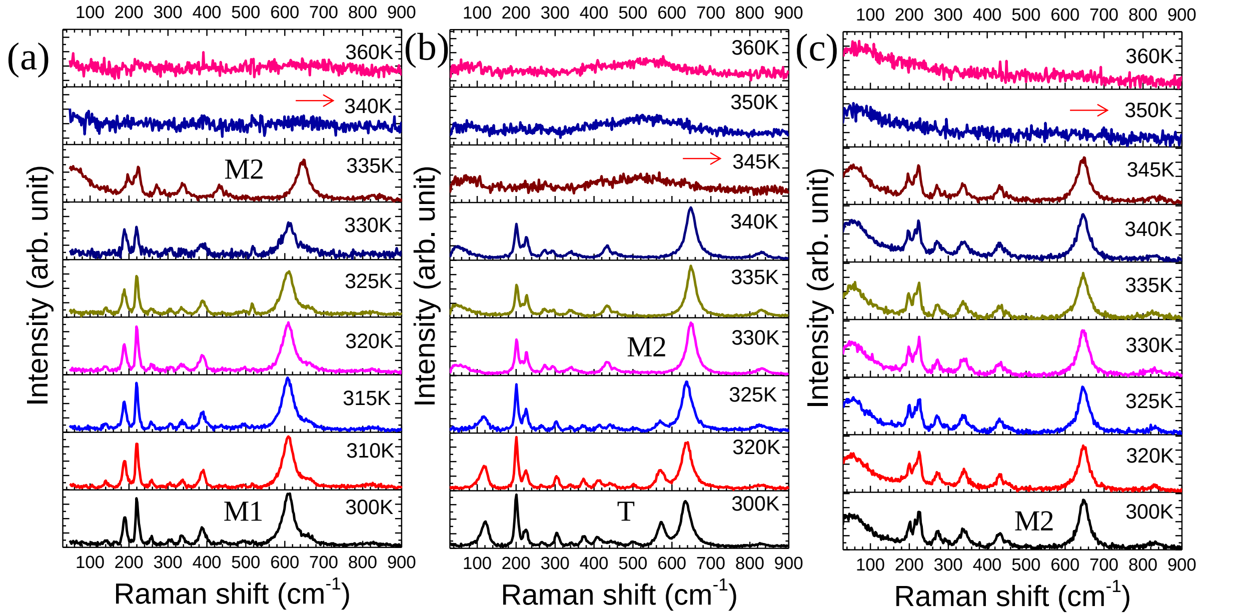

Fig. S4 Raman spectra of VO_2 microcrystals of samples (a) S1, (b) S2 and (c) S3b with increasing temperature. Arrow denotes the corresponding transition temperature.

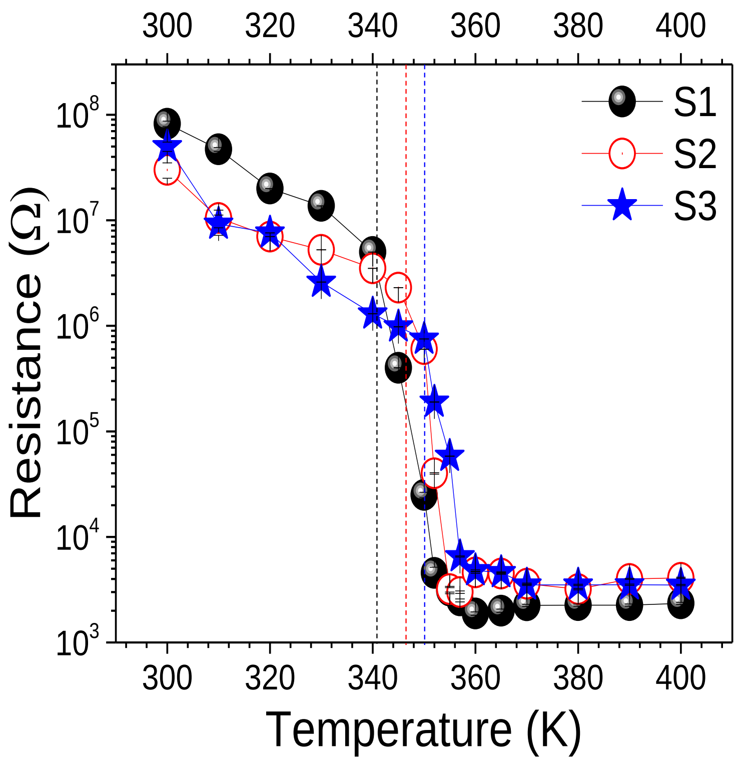

Fig. S5 Resistance measurement for the VO_2 samples S1, S2, and S3b as a function of temperature showing a drop in resistance of three to four orders indicating metal insulator transition (shown by vertical dashed lines).

References:

1. Garcıa-Escarzaga, A., Moncayo, S., Gutierrez-Zugasti, I., Gonzalez-Morales, M. R., Martın-Chiveletc, J., Caceres, J. O. & Anal. J. *At. Spectrom*. **30**, 1913-1919 (2015).
2. CRC Handbook for Chemistry and Physics, 62nd Edition, 1981-1982.
